# Bacteria endocarditis consolidation with vertebra bone tuberculosis: a case report

**DOI:** 10.1186/s12879-016-2168-9

**Published:** 2017-01-06

**Authors:** Yan Liu, Xiaoming Wang, Zhibin Wang, Yongsheng Zhu, Liying Zhang, Xiaoli Li, Rong Xu, Wei Ge

**Affiliations:** 1 Department of Geriatrics, Xijing Hospital, Fourth Military Medical University, Changle West Road #127, Xi’an, Shaanxi Province 710032 People’s Republic of China; 2 Department of ultrasound diagnosis, Xijing Hospital, Fourth Military Medical University, Xi’an, Shaanxi Province 710032 People’s Republic of China; 3 Department of Pathology, Xijing Hospital, Fourth Military Medical University, Xi’an, 710032 Shaanxi Province China; 4 Department of Geriatrics, Weinan Central Hospital, Weinan, 714000 Shaanxi Province China

**Keywords:** Bacteria endocarditis (BE), Thoracic vertebra tuberculosis, Viridans streptococcus

## Abstract

**Background:**

The clinical features of bacteria endocarditis became atypical when consolidated with other conditions such as tuberculosis (TB). Especially, the symptoms of bacteria endocarditis (BE) which were hidden behind the TB fever often lead to misdiagnosis and missed diagnosis.

**Case presentation:**

A 56-year-old male with thoracic vertebra bone TB history presented with low-grade fever, shortness of breath and cardiac souffle. After conventional antibiotic therapy and strengthen anti-tuberculosis treatment condition did not be improved. Further inspection, there were bacteria endocarditis with the vegetation across the mitral valve. But the other valves were not involved. He was treated with intravenous penicillin for 4 weeks in all including during surgery, and following with oral antibiotic for another 2 weeks. The patient improved clinically eventually.

**Conclusion:**

It is the first reported case of isolated thoracic vertebra tuberculosis with valve endocarditis caused by streptococcus viridans and was successfully managed by combination therapy of internal medicine and surgery. It was suggested in tuberculosis patients, the possibility of bacterial endocarditis should be considered when came into fever and unexplained cardiac soufflé (in tuberculosis patients).

## Background

The clinical manifestation of bacteria endocarditis (BE) became atypical due to the abusing of wide-spectrum antibiotics and the mutation of the micro-organism, especially consolidation with other conditions which could cause body immunity decline such as TB, long-term use of glucocorticoids, HIV infection, intravenous drug abuse [1, 2], and so on. As everyone knows, TB, a systemic disease which can cause malnutrition and further effect the cellular immune functions remarkably. TB is known to affect the pericardium [3], myocardium [4], and valvular structure of the heart [5–7] as well as other organs of the body [8]. The case of bone tuberculosis consolidation with BE has not been reported so far. In this report, the malnutrition thoracic vertebra bone TB patient with constantly fever and cardiac soufflé, he was ultimately diagnosed as BE by transesophageal echocardiography, blood culture, and was cured eventually by combination treatment of internal medicine and surgery.

## Case presentation

A 56-year-old man with low fever, shortness of breath, drenching night sweats, anorexia and a 10 kg weight loss was admitted to the Gerontology department in February, 2015. 40 days before hospital he suffer a low-grade fever which appeared intermittently and high up only to 38 °C, accompanied with fatigue, short of breath, night sweat, without shiver, cough, rash, twinge of arthritis, and so. Tracing back the history, 5 months ago, he was diagnosed with thoracic vertebra TB and he was still under anti-tuberculous chemotherapy. The cardiac examination history 5 months ago, he already had cardiac soufflé but without further diagnosis and treatment. After outpatient clinic strengthening anti-tuberculosis treatment for 40 days, the above symptoms had not been any improved. After coming to the hospital, on physical examination, he appeared feeble and malnutrition. The temperature was 37.6 °C, heart rate 95 beats/min, blood pressure 130/70 mmHg, respiratory rate 18 breaths/min and oxygen saturation 98% on 2 L nasal prongs. Cardiac examination revealed a moderate S3, a grade III/VI aortic systolic ejection murmur, without signs of congestive heart failure. Abnormal laboratory investigations included a normochromic anemia with a hemoglobin level of 99 g/L (normal level 140 g/L to 160 g/L), a hypoproteinemia with albumin level of 30 g/L (normal value 40 g/L to 50 g/L) and elevated inflammatory markers erythrocyte sedimentation rate (ESR): 60 mm/hr (normal value 0 mm/hr to 10 mm/hr) and C-reactive protein level 62.7 mg/L (normal level 0 mg/L to 8 mg/L) (Table 1). HIV in serum were detected to be negative by ELISA and other routine laboratory tests were within the normal range including the white blood cell (WBC) count of 7.44 × 10^9^/L (normal values 4 × 10^9^/L to 10 × 10^9^/L), neutrophils 5.76 × 10^9^/L (normal values 2 × 10^9^/L to 8 × 10^9^/L).

**Table 1:** Clinical parameters of the patient

Clinical Parameters	Heart rate (beats/min)	Blood pressure (mmHg)	Respiratory rate (breaths/min)	Hemoglobin (g/L)	Albumin (g/L)	ESR (mm/hr)	C-reactive protein (mg/L)
The patient	95	130/70	18	99	30	60	62.7
Normal value	60–100	140–90/90–60	16–20	140–160 (male) 130–150 (female)	40–50	0–15 (male) 0–20 (female)	<10

**Fig. 1:**
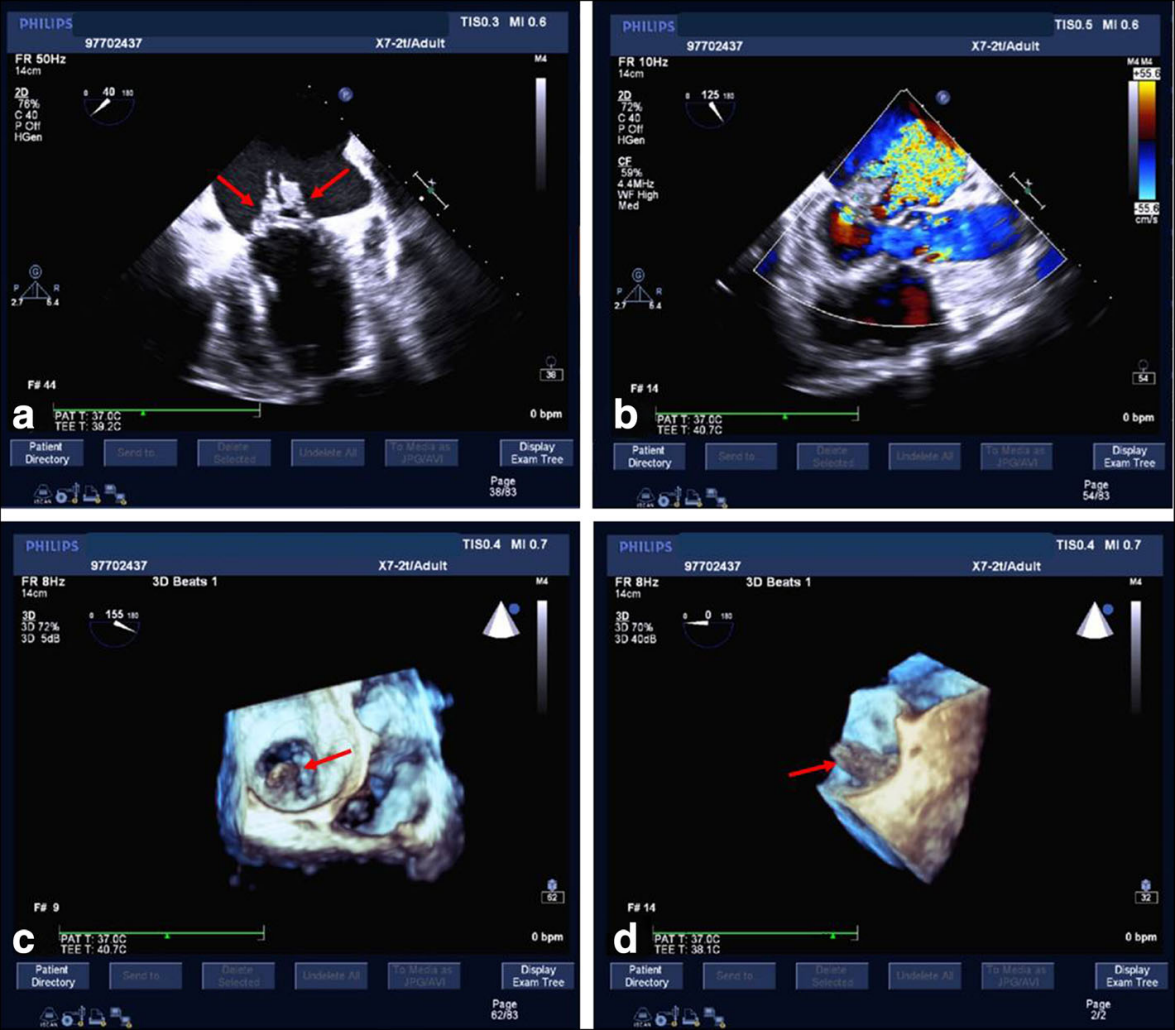
Images of mitral valve by 2D and real-time 3D transesophageal echocardiography (TEE). **a** Medium to high-echoic vegetations adhering to anterior and posterior mitral leaflets and part of the posterior mitral leaflet prolapsing into left atrium in systole by 2D TEE. **b**. Severe mitral insufficiency and perforation with regurgitation; **c** and **d**. A bird-eye view and a lateral view of mitral valve vegetations from left atrium by real-time 3D TEE

**Fig. 2:**
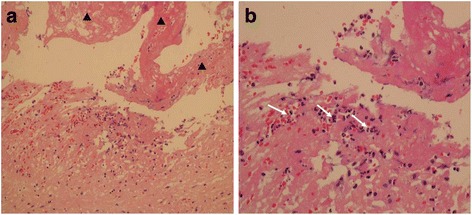
Result of the tissue pathology of cardiac mitral valve. Mitral valve sample is stained by hematoxylin-eosin staining (HE) and shows fibrinoid necrosis (▲) with neutrephil infiltration (→) (HE: **a** ×200; **b** ×400)

The blood culture was performed when the body temperature was high up to 38 °C. He was earlier empirically treated with intravenous moxifloxacin hydrochloride 0.4 g once a day. The transthoracic echocardiography (TTE) and transesophageal echocardiography (TEE) was performed on the 3rd day in hospital (Fig. 1). Images of mitral valve by 2D and real-time 3D TEE show medium to high-echoic vegetations adhering to anterior and posterior mitral leaflets and part of the posterior mitral leaflet prolapsing into left atrium in systole, and severe mitral insufficiency and perforation with regurgitation 2D TEE. A bird-eye view and a lateral view of mitral valve vegetations from left atrium by real-time 3D TEE. And the result of the pathology reconfirmed: acute inflammation of the chronic cardiac valve disease (Fig. 2). Two days later, the blood culture result indicated that he was infected with viridans streptococcus. Finally intravenous penicillin G 2400 kU every 4 h (q4h) was used according to the drug sensitive experiment of the blood culture.

Then he was transferred to the chest surgery department for surgery until the temperature was normal, and treated with intravenous penicillin G the same as before for another 2 weeks. Oral antibiotics amoxicillin capsules 0.5 g q8h was recommended for another 2 weeks post-discharge in order to prevent recurrence.

## Discussion

The patient had the bone tuberculosis and heart murmur history for 5 months, low-grade fever but no hyperpyrexia, WBC in the normal range, and highly suspected tuberculosis resurgence. However, during adjustment to strengthen the antituberculosis treatment, the clinical curative effect was not improved. Therefore, the other diseases caused a series of clinical problems, such as fever, heart murmur should be considered. The result of blood culture, heart TEE, and the surgical pathological examination, finally confirmed for bacterial endocarditis, rather than tuberculous endocarditis. He was treated with intravenous penicillin for 4 weeks, then following with oral antibiotic for another 2 weeks, and finally clinically improved and discharged back to the home. Three months later, the patient was asymptomatic without any complications and got much better, a repeat TEE did not show any mitral valve vegetation. In this patient case, the differential diagnosis of bacterial endocarditis and tuberculous endocarditis is difficult, easy to be misdiagnosed.

How to discriminate TE and BE? BE is infected by bacterial, with strong virulence and serious general toxic symptom showing hyperpyrexia, progressive anemia, splenomegaly, WBC and ESR increasing obviously. While TE is infected by mycobacterium tuberculous, with relatively weak virulence, light general toxic symptom including low-grade fever, fatigue. However, the patients in this case, accompanied by bone TB, the clinical manifestation is not so significantly typical. So, the possibility of BE infection should be considered when anti-TB treatment was non-effective for TB patients with heart murmur, even with low-grade fever and normal WBC.

In recent decades, the epidemiological characteristics and the clinical features of BE has changed because of the widely use of broad-spectrum antibiotics and the increasing patients with cardiac surgery and heart intervention operation [9]. BE remains as a life threatening disease with high morbidity and mortality. The prophylaxis, diagnosis, and treatment are still a major challenge in clinical. Even though, long duration of fever is still the most common clinical manifestation of BE. In additional, heart murmur is also another common performance secondary to the fever and it cannot be neglected in this case. In view of this, when the anti tuberculosis treatment effect is not so good, the possibility of suffering from bacterial endocarditis be suspected.

The two main standards following the Duke diagnosis criteria: the positive rate of blood culture and the positive findings of echocardiography [10, 11]. The neoplasm formation is the most elementary pathological change of BE [12]. And it most commonly affect mitral valve, secondly aortic valve, in addition also affect pulmonary valve [13]. Accordingly TEE was conducted decisively to help us to diagnosis in this case. At the same time, it also provides convenience for the subsequent surgical treatment, greatly reducing the mortality, improve the quality of life [9, 14].

## Conclusion

Clinical doctors should pay more attention to BE, especially the BE patients accompanied with susceptible factors, such as tuberculosis, HIV, intravenous drug abuse and so on. Early detection and diagnosis [15], timely and adequate antimicrobial therapy, and grasping the optimal and principle for surgery meantime, are the keys to cure patients with BE.

## Abbreviations

BE: Bacteria endocarditis
ESR: Erythrocyte sedimentation rate
TB: Tuberculosis
TEE: Transesophageal echocardiography
TTE: Transthoracic echocardiography
WBC: White blood cell
